# EBUS-TBNA for Diagnosis and Staging of Lung Cancer: A Retrospective Regional Analysis Integrating Clinical and Molecular Data (EXPoSURE Score)

**DOI:** 10.3390/jcm14176179

**Published:** 2025-09-01

**Authors:** Gabriela Marina Andrei, Natalia Motaș, Virginia Maria Rădulescu, Nina Ionovici, Marius Bunescu, Daniela Luminița Zob, Viorel Biciușcă, Florentina Dumitrescu, Eugenia Andreea Marcu, Ramona Cioboată, Mihai Olteanu

**Affiliations:** 1 Department of Internal Medicine—Pneumology, Faculty of Medicine, University of Medicine and Pharmacy of Craiova, 200349 Craiova, Romania; gabriela.andrei@umfcv.ro (G.M.A.); mihai.olteanu@umfcv.ro (M.O.); 2 Department of Thoracic Surgery II, Faculty of Medicine, “Carol Davila” University of Medicine and Pharmacy, 022328 Bucharest, Romania; 3 Department of Medical Informatics and Biostatistics, Faculty of Medicine, University of Medicine and Pharmacy of Craiova, 200349 Craiova, Romania; 4 Department of Occupational Medicine, Faculty of Medicine, University of Medicine and Pharmacy of Craiova, 200349 Craiova, Romania; 5 Department of Medical Oncology II, “Prof. Dr. Al. Trestioreanu” Institute of Oncology, 022328 Bucharest, Romania; 6 Department of Infectious Diseases, Faculty of Medicine, University of Medicine and Pharmacy of Craiova, 200349 Craiova, Romania

**Keywords:** lung cancer, EBUS-TBNA, mediastinal staging, molecular markers, PD-L1, EXPoSURE score, occupational exposure

## Abstract

**Background/Objectives**: Lung cancer remains the leading cause of cancer-related mortality worldwide, with a high proportion of cases diagnosed at advanced stages. Accurate mediastinal staging is essential to guide optimal therapeutic decisions. This study aimed to evaluate the diagnostic performance of endobronchial ultrasound-guided transbronchial needle aspiration (EBUS-TBNA) and to develop a composite clinical–molecular score (EXPoSURE) for risk stratification. **Methods**: A retrospective study was performed that included 131 patients diagnosed with lung cancer between December 2023 and December 2024 at a regional oncology center in Oltenia, Romania. All patients underwent bronchoscopy and EBUS-TBNA using a standardized protocol. Clinical, pathological, and molecular data were collected to assess diagnostic yield, staging performance, and the association with molecular markers. The EXPoSURE score integrated PD-L1, p63, EGFR status, comorbidities, histological type, and TNM stage. **Results**: EBUS-TBNA provided a conclusive diagnosis in 91.6% of cases, with a low rebiopsy rate of 8.4% and no requirement for mediastinoscopy. Most patients (68%) were diagnosed at stage IV. PD-L1, p63, and EGFR expression showed no significant correlation with TNM stage, while the EXPoSURE score demonstrated promising stratification capability. Occupational exposure appeared to influence disease severity in some subgroups, although further validation is needed. **Conclusions**: EBUS-TBNA is a valuable, safe, and effective approach for minimally invasive diagnosis and mediastinal staging of lung cancer. The proposed EXPoSURE composite score may contribute to a multidimensional risk assessment, supporting more tailored management strategies and warranting prospective validation.

## 1. Introduction

Lung cancer remains the leading cause of cancer death worldwide. Incidence and mortality have declined in the United States, while cases worldwide continue to increase. Risk factor modification and screening are essential for improving survival in patients with lung cancer [1]. Although smoking is the main risk factor, other risk factors have been associated with the etiology of lung cancer [2], including occupational exposure to carcinogens, especially radon, passive smoking, coal burning, hormonal modifications, infections with Mycobacterium Tuberculosis and Human Papilloma Virus (HPV), or genetic factors [3].

The histopathological classification according to the World Health Organization (WHO) revised in 2021 highlights non-small cell lung cancer (NSCLC), such as adenocarcinomas, squamous cell carcinomas, and large cell carcinoma; small cell lung cancer (SCLC); and neuroendocrine tumors of the lung, i.e., typical low-grade carcinoids and atypical intermediate-grade carcinoids, but also neuroendocrine carcinomas (LCs). Important advances in the knowledge of genetic mutations in lung cancer and in the development of targeted therapies against these mutations have led to significant improvements in patient survival [4]. Currently, testing for multiple additional driver gene mutations or fusions (e.g., ROS1, BRAF, NTRK1-3, RET, KRAS, and MET) in addition to EGFR and ALK and testing for the immune biomarker programmed death ligand 1 (PD-L1) is recommended [5].

Another classification is the TNM classification, which is a standardized system used to describe the stage of lung cancer and to guide treatment and prognosis. It is based on three descriptors: T-primary tumor, N-lymph nodes, and M-metastases [6]. The ninth edition of the TNM classification for lung cancer, set to be implemented in January 2025, addresses this requirement. It is based on a comprehensive international database, input from multiple disciplines, and in-depth statistical analyses. Notable updates in the ninth edition include the validation of significant changes to the T component introduced in the eighth edition, a revised approach to categorizing the N2 component after exploring alternative classification methods, and further refinement of the M component. As a result, the TNM combinations within stage groups, particularly stage groups IIA, IIB, IIIA, and IIIB, have been reordered [7].

Bronchoscopy is the most important tool for diagnosing lung cancer, carried out by taking a biopsy and performing an anatomopathological examination [8]. For over 150 years, bronchoscopy, particularly flexible bronchoscopy, has been used to diagnose lung disease. Recent studies have shown the ability to perform biopsies at the periphery of the lung, safely avoid vascular structures, and diagnose a tumor under direct visualization [9]. Currently, another indispensable tool is endobronchial ultrasound-guided transbronchial needle aspiration (EBUS-TBNA), which has become an indispensable tool in the diagnosis and staging of lung cancer [10]. It is also used to diagnose mediastinal diseases. Over the past 10 years, this procedure has undergone significant technical advancements, with new tools developed to enhance tissue sampling and improve diagnostic accuracy, including elastography, various needle types, and more recently, mini forceps and cryobiopsy [11]. Mediastinoscopy was historically regarded as the gold standard for staging mediastinal lymph nodes; however, in recent years, endobronchial ultrasound-guided (EBUS) fine-needle aspiration (FNA) has become the preferred method of care [12]. EBUS-FNA is also effective in evaluating N1 disease and providing sufficient tissue for tumor genomic analysis, which can assist in guiding treatment decisions [13].

The future of lung cancer treatment is expected to be marked by significant innovations, especially in the fields of targeted therapies, immunotherapy, and advanced diagnostic technologies. Therefore, great emphasis is placed on histopathological and immunohistochemical diagnosis, accurate staging, and genetic testing [14].

Despite international recommendations favoring EBUS-TBNA for mediastinal staging in lung cancer, its implementation in Romanian medical centers has been hindered by barriers such as limited training opportunities, lack of equipment standardization, and insufficient multidisciplinary collaboration. Consequently, many patients still undergo invasive surgical procedures or incomplete diagnostic assessments. In the Oltenia region, there is no published experience describing routine EBUS-TBNA use. Similar barriers are reported in other low- and middle-resource regions, illustrating disparities in EBUS adoption and the risk of delayed treatment planning [10,11,12,13,14,15]. By systematically analysing its diagnostic accuracy and its influence on therapeutic decision-making, the current study aims to provide a framework for expanding minimally invasive staging practices in similar regional settings. In Eastern Europe, disparities in access and capacity for EBUS-TBNA remain significant, with marked variability in training opportunities and waiting times. Romania currently lacks official EBUS training centres, and there is a clear east–west divide in access to essential diagnostic procedures, including EBUS-TBNA, PET-CT, and molecular testing [16,17,18]. In this context, the main objective of the present study is to evaluate the diagnostic and staging value of bronchoscopy and EBUS-TBNA in lung cancer by analysing the correlations between histopathological subtypes and the expression of immunohistochemical and molecular markers (PD-L1, p63, EGFR) in relation to TNM clinical staging. This regional gap in minimally invasive mediastinal staging provided the rationale for a systematic evaluation of EBUS-TBNA performance and the development of a multidimensional clinico-molecular stratification tool.

In parallel, composite prognostic indices that combine clinical and inflammatory markers—most notably the lung immune prognostic index (LIPI)—have shown consistent prognostic value in NSCLC cohorts treated with immune checkpoint inhibitors and have undergone external validation and meta-analytic confirmation, supporting the use of multidimensional approaches such as our EXPoSURE score [19,20,21].

Also, as a secondary objective, the study aims to investigate the relationship between occupational exposure and the clinical, histopathological, and molecular profile of the included patients in order to highlight possible additional influencing factors on disease severity.

We hypothesized that EBUS-TBNA would achieve a high diagnostic yield and provide accurate mediastinal staging in lung cancer patients from the Oltenia region, despite the constraints of a resource-limited setting. Furthermore, we assumed that integrating histopathological, immunohistochemical, molecular, and clinical parameters into a composite clinical–molecular score (EXPoSURE) could stratify patients according to disease severity. The primary aim of this study was to evaluate the diagnostic and staging performance of EBUS-TBNA in this population and to develop and internally validate the EXPoSURE score. Our main findings indicate a diagnostic yield of 91.6% for EBUS-TBNA and a significant association between higher EXPoSURE scores and advanced TNM stages, supporting its potential utility in clinical decision-making.

## 2. Materials and Methods

The present study is a retrospective, observational study conducted from December 2023 to December 2024; it included 131 consecutive patients diagnosed with lung cancer. Patients were evaluated and diagnosed in a regional multidisciplinary center that specializes in pneumology and oncology, representing, at the time of the study, the only center in the Oltenia region that consistently performs EBUS-TBNA procedures integrated with the oncology diagnostic algorithm.

The histopathological diagnosis was established on the basis of the fragments collected by minimally invasive methods: conventional bronchoscopy with or without ultrasound-guided transbronchial puncture (EBUS-TBNA), depending on the clinico-radiological particularities of each case. Histopathological and molecular diagnosis was performed in all cases by minimally invasive methods using conventional bronchoscopy and/or EBUS-TBNA. Collection of tumor and/or lymph node material was possible for all patients, and rebiopsies were performed in cases where material was insufficient.

The study was approved by the Ethics Commission of the University of Medicine and Pharmacy of Craiova, in collaboration with the Centre of Oncology and Radiotherapy “Sfântul Nectarie” of Craiova (Opinion no. 26/720/1/20.07.2023) and was conducted in accordance with the Declaration of Helsinki of the World Medical Association. All patients were informed about the nature of the study, the protection of personal data and the right to withdraw at any time, regardless of the reason or the evolution of their health condition. Participation in the study was on the basis of written informed consent only.

The sample size justification was performed using G*Power 3.1.9.7 software. For testing associations between histopathological subtypes and clinical–molecular markers or TNM stage, a sample of at least 126 patients was estimated, considering a chi-square test with 4 groups (G1a–G3), a moderate–low effect size (w = 0.25), a probability α of 0.05, and a statistical power (1 − β) of 0.80. The group of 131 patients included in the study is adequate to detect statistically significant differences, even in the presence of more subtle effects.

### Inclusion and Exclusion Criteria

Patients who met the following criteria were included in the study:

- Histopathological confirmation of a lung cancer;
- Stable general clinical condition that allowed bronchoscopy with or without EBUS-TBNA;
- Written consent for participation in the study and use of medical data for scientific purposes.

The exclusion criteria were as follows:

- Severe coagulation disorders;
- Major mental illness;
- Clinical or imaging suspicion of lung cancer without histological confirmation.

All bronchoscopic and EBUS-TBNA procedures were performed by experienced physicians using a flexible videobronchoscope (PENTAX Medical EB15-J10, PENTAX Medical, Akishima-shi (Tokyo), Japan) connected to a PENTAX EPK-i7010 video processor and a compatible ARIETTA 850 ultrasound system. In selected cases, depending on equipment availability, a Pentax EB-1970UK videobronchoscope with EBUS capability was also utilized. For lymph node aspirations, 22G needles (Echotip Ultra COOK ECHO-HD-22-EBUS-P (Cook Medical, Bloomington, IN, USA) or equivalent Olympus (Shinjuku City, Japan)/Pentax models) were employed. Procedures were carried out under local anesthesia with xylocaine and mild intravenous sedation.

The ultrasound characteristics of the targeted lymphadenopathies were documented following international guidelines, and sampling order was determined based on prior thoracic CT imaging. Typically, punctures started with the most distant pathologic lymph node station relative to the primary pulmonary tumor, progressing toward it. Priority was given to stations 4R, 4L, and 7 for N2 disease, and stations 10R and 11L for N1 involvement, depending on tumor localization. When multiple lymph node stations within the N2 descriptor (according to the ninth TNM edition) were involved, samples were obtained from each station to ensure complete staging. For each sampled station, a minimum of three core tissue fragments was collected to maximize diagnostic accuracy.

Overall, EBUS-TBNA achieved a conclusive diagnosis in most cases (91.6%, 95% CI: 85.8–95.8%), with a low rebiopsy rate of 8.4% (95% CI: 4.3–14.4%; *n* = 11). The combined use of bronchoscopy and EBUS-TBNA provided adequate tumor and lymph node material for histopathological, immunohistochemical, and molecular evaluation. Furthermore, lymph node staging was effectively performed using EBUS-TBNA without the need for mediastinoscopy in any patient.

Processing of the biopsy fragments was performed in the accredited Pathology Laboratory according to standard fixation and staining protocols (hematoxylin–eosin). The histological type was determined according to WHO classification 2021.

For immunohistochemistry, antibodies were specific for the following:

- p63—to identify squamous cell carcinoma;
- PD-L1—expression evaluated semi-quantitatively by TPS (tumor proportion score) method;
- Ki-67—as cell proliferation marker (expression in % positive tumor cells).

EGFR mutation testing was performed by real-time PCR with the determination of exons 18, 19, 20, and 21. Tests for ALK and ROS1 were not available in all cases and were not included in the final analysis.

PD-L1 expression was evaluated semi-quantitatively using the tumor proportion score (TPS) method, with an FDA-approved assay (clone and kit details available upon request), following the manufacturer’s instructions. p63 status was determined by immunohistochemistry using a validated diagnostic antibody (Clone SP263—Ventana), interpreted according to standard morphological and staining criteria. EGFR mutation testing was performed by real-time PCR using the cobas^®^ DNA Sample Preparation Kit (Roche Diagnostics, Mannheim, Germany) for DNA extraction, followed by the cobas^®^ EGFR Mutation Test v2 (Roche Diagnostics, Mannheim, Germany) on the Cobas Z 480 platform, targeting exons 18, 19, 20, and 21, in accordance with the manufacturer’s guidelines.

Primary data were first organized and categorized for statistical analysis using Microsoft Excel 2021 (Microsoft Corp., Redmond, WA, USA). Statistical analyses were performed using IBM SPSS Statistics, version 26.0 (IBM Corp., Armonk, NY, USA). The normality of continuous variables was assessed using the Shapiro–Wilk test, supplemented by visual inspection of scatter plots. Continuous variables that did not follow a normal distribution were expressed as median values with interquartile ranges (IQRs). Nominal and ordinal variables were summarized as absolute and relative frequencies (%). Associations between nominal variables were tested using the chi-square test, with Monte Carlo simulation applied when cell counts were <5. Differences between groups for continuous variables were examined using the Kruskal–Wallis test, and ordinal variables were assessed using symmetry tests. For each hypothesis test, effect sizes (Cramer’s V or odds ratios) and corresponding 95% confidence intervals were reported alongside *p*-values. No formal corrections for multiple testing were applied, as the analyses were primarily exploratory and aimed at identifying potential associations for further validation. A *p*-value <0.05 was considered statistically significant.

Analysis included correlations between TNM stage and histopathological types, as well as the association of molecular markers with histological distribution and clinical severity. The influence of occupational exposure in the context of the secondary endpoint was also analyzed.

The discriminatory ability of the EXPoSURE score was further assessed using receiver operating characteristic (ROC) curve analysis. Two reference outcomes were considered: (i) stage IV disease and (ii) an alternative definition of advanced disease grouping TNM stages III–IV versus I–II. The area under the curve (AUC) with 95% confidence intervals was calculated for each scenario. To evaluate the stability of the predictive performance and reduce the risk of overfitting, internal validation was performed using bootstrap resampling with 1000 iterations, and optimism-corrected AUC values were reported.

**Figure 1:**
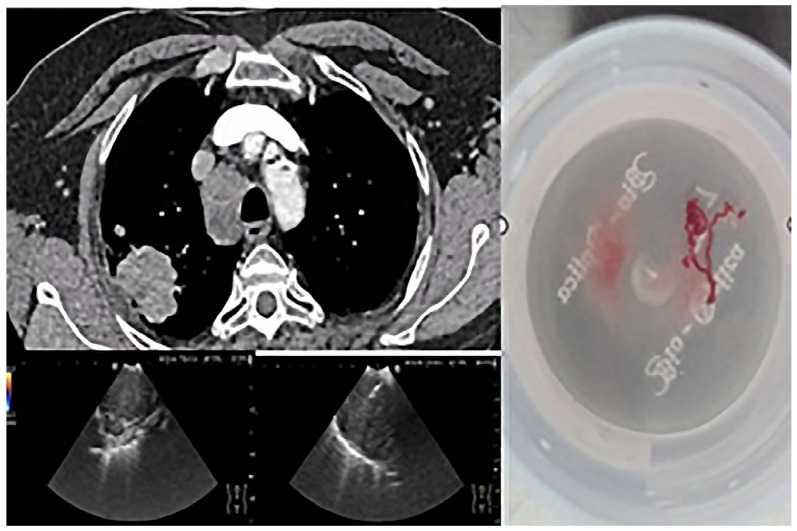
EBUS-TBNA in a 69-year-old ex-smoker male patient with no endobronchial tumor expression. Local anesthesia and mild sedation were used for transbronchial needle aspiration from 4R lymph node station, as shown in the thoracic CT section. Small cell carcinoma was confirmed and staged (N descriptor) in the same procedure. Immunohistochemical profile: TTF1-positive, SYN-positive, ki67-positive 98% in tumor cells.

The importance of using EBUS-TBNA in this study is illustrated in Figure 1, which shows a lymph node puncture procedure in a patient with non-small cell carcinoma. In this example, the targeted lymph node station (4R) is annotated in the image. Sampling was performed using a 22G needle, and a scale bar has been added to provide spatial reference for the structures visualized. This technique allowed material suitable for complete characterization to be obtained without resorting to invasive surgical methods.

## 3. Results

### 3.1. General Demographic and Histopathological Characteristics

Of the total 131 patients included in the study, 53 (40.5%) were female and 78 (59.5%) were male. The distribution according to background did not differ significantly between the two groups (χ^2^ = 1.060; *p* = 0.303, Cramer’s V = 0.09, 95% CI: 0.00–0.21), with 41.2% of patients coming from rural and 58.8% from urban areas. Smoking status was more frequent among men, but without statistical significance (χ^2^ = 2.869; *p* = 0.090; Cramer’s V = 0.15, 95% CI: 0.00–0.28). Regarding comorbidities, significant differences by sex were observed (χ^2^ = 16.910; df = 4; *p* = 0.002, Cramer’s V = 0.36, 95% CI: 0.18–0.50), as detailed in Table 1.

Although the prevalence of smoking was higher among male patients (44.3% vs. 24.4% in women), this difference was not statistically significant (χ^2^ = 2.869; *p* = 0.090, Cramer’s V = 0.15, 95% CI: 0.00–0.28); however, the trend suggests a potential association that could be explored in future studies with a larger sample.

In terms of comorbidities, there were significant differences by sex (χ^2^ = 16.910; df = 4; *p* = 0.002, Cramer’s V = 0.36, 95% CI: 0.18–0.50). Asthma was reported exclusively in women (6.1%), while diabetes was more common in men (14.5% compared with 3.1%). COPD was present in 15.3% of women and 21.4% of men, and heart disease in 9.2% and 13.0%, respectively. A total of 17.6% of patients had no comorbidities. Males showed a higher frequency of chronic obstructive pulmonary disease (COPD) and diabetes, while asthma was identified exclusively in females. This different distribution may reflect different risk factors and gender-specific biological or behavioral influences.

Occupational exposure to risk factors was reported in 57.3% of patients, with no significant gender differences (χ^2^ = 0.015; *p* = 0.902, Cramer’s V = 0.01, 95% CI: 0.00–0.13).

Patient age ranged from 40 to 85 years, with a mean of 62.82 years (±12.67 years). The 95% confidence interval for the mean was [60.63–65.01] years. The adjusted mean (5% trimmed mean) was 62.87 years, very close to the median value (63 years), suggesting a symmetrical distribution of the data. This observation is also supported by the skewness coefficient close to zero (skewness = −0.077), indicating a balanced distribution, with no significant extreme values distorting the descriptive analysis.

Molecular biomarker expression analysis revealed the following results: out of 131 patients, 84 (64.1%) were p63-positive and 47 (35.9%) were p63-negative. Regarding PD-L1, 72 patients (55.0%) had positive expression, while 59 (45.0%) were negative.

Evaluation of EGFR mutations showed that the majority of patients (*n* = 112; 85.5%) did not have mutations. Of the 19 patients (14.5%) with EGFR mutations, the distribution was as follows: exon 19—9 cases (6.9%), exon 21—5 cases (3.8%), exon 20—3 cases (2.3%), and exon 18—2 cases (1.5%).

The distribution of cases by histopathological diagnostic groups was G1a—45 patients (34.4%), G1b—29 patients (22.1%), G2—30 patients (22.9%), and G3—27 patients (20.6%).

The data in Table 1 provide a comprehensive picture of the socio-demographic and clinical characteristics of patients, highlighting both similarities and gender differences that may have implications for preventive and therapeutic approaches.

**Figure 2:**
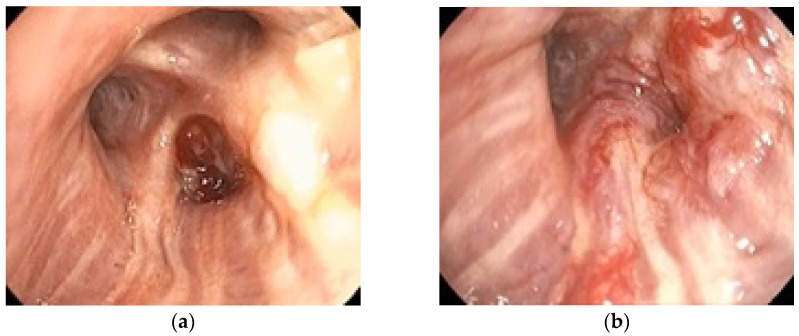
Adenocarcinoma of the dorsal segment of the right upper lobe in a 72-year-old male ex-smoker, well vascularized, with distal invasion of the lobar bronchus and significant stenosis of its segmental branches due to extrinsic compression and mucosal infiltration. Rapid growth in one month with lack of any oncologic treatment: (**a**) 12 December 2023; (**b**) 16 January 2024.

To illustrate the clinical relevance of early endoscopic evaluation, Figure 2 shows the rapid progression of a case of bronchial adenocarcinoma with notable changes occurring within one month. The tumor diameter increased from 28 mm in December 2023 to 45 mm in January 2024, over a one-month interval without oncologic treatment. These quantitative measurements highlight the aggressive nature of the disease and the importance of timely staging. This example supports the need for accurate staging, achievable by bronchoscopy and EBUS.

### 3.2. Analysing the Distribution of Risk Factors by Background

To complete the characterization of the group, the distribution of smoking status, comorbidities, and occupational exposure according to the background (Table 2) was also assessed. The proportion of smokers was comparable between rural and urban patients, 38 (29.0%) and 52 (39.7%), respectively, with no statistically significant differences (χ^2^ = 0.12, *p* = 0.730; Cramer’s V = 0.030, 95% CI: 0.000–0.120). Similarly, the presence of comorbidities, including asthma, chronic obstructive pulmonary disease (COPD), heart disease, and diabetes, was relatively evenly distributed between the two settings, with no significant associations (χ^2^ = 3.03, *p* = 0.391; Cramer’s V = 0.152, 95% CI: 0.000–0.247).

In terms of occupational exposure, the proportions of exposed patients were close in rural and urban areas (31 versus 44 patients), with no significant differences (χ^2^ = 0.00, *p* = 0.976; Cramer’s V = 0.001, 95% CI: 0.000–0.050).

Thus, the data indicate that the background did not significantly influence smoking status, the presence of comorbidities, or occupational exposure in the study group, in agreement with the observations on the distribution of sex and these factors in the previous analysis (Table 1).

### 3.3. Distribution of Smoking Status and Comorbidities by Occupational Exposure

In the study group (*n* = 131 patients), the analysis of the distribution of smoking status according to occupational exposure revealed a higher frequency of smokers among occupationally exposed patients (41.2%) compared to non-exposed patients (27.5%). The proportion of non-smokers was similar between the two groups: 16.0% in the exposed group and 15.3% in the non-exposed group.

In order to assess the association between occupational exposure and smoking status, the Pearson chi-square test, which is appropriate for this analysis (2 × 2), was applied, as none of the cells had expected frequencies below 5 (the minimum expected frequency value was 20.34). The test indicated an χ^2^ value = 0.892, with 1 degree of freedom, and a statistical significance of *p* = 0.346, Cramer’s V = 0.082, and 95% CI [0.000–0.200], suggesting the absence of a significant association between the two variables.

Regarding comorbidities, their distribution between occupationally exposed and non-exposed patients was as follows: 6.9% of non-exposed patients and 10.7% of exposed patients had no comorbidities; asthma was more frequent in the non-exposed group (3.8% vs. 2.3%); chronic obstructive pulmonary disease (COPD) was more common in unexposed patients (23.7% vs. 13.0%); heart disease had a similar distribution (11.5% vs. 10.7%); and diabetes was reported in 7.6% of unexposed and 9.9% of exposed patients.

The Pearson chi-square test applied for the association between occupational exposure and type of comorbidity was interpretable, as there were no expected frequencies below 5 (minimum 8.12). The result χ^2^ = 3.515, with 4 degrees of freedom, revealed a lack of statistically significant association (*p* = 0.491, Cramer’s V = 0.161, 95% CI [0.000–0.260]), indicating that the distribution of comorbidities did not differ according to occupational exposure in the analyzed group (Table 3).

### 3.4. The Association Between Smoking Status and the Presence of Comorbidities

Analyzing the distribution of comorbidities according to smoking status reveals statistically significant differences (Table 4). Thus, among non-smokers, 5 cases without comorbidities, 8 with asthma, 14 with chronic obstructive pulmonary disease (COPD), 11 with heart disease, and 3 with diabetes were reported. In the smoking group, 18 patients had no comorbidities, none had asthma, 34 had COPD, 18 had heart disease, and 20 had diabetes.

The Pearson chi-square test indicated a statistically significant association between smoking status and type of comorbidity (χ^2^ = 22.797, df = 4, *p* < 0.001, Cramer’s V = 0.470, 95% CI [0.330–0.570]). Although one of the cells had an expected frequency below 5, the validity of the test was supported by applying Monte Carlo correction, which confirmed the significance of the results (Monte Carlo *p* = 0.000, 99% confidence interval: 0.000–0.000).

This rigorous analysis confirms a strong association between smoking status and the presence of specific types of comorbidities, particularly for chronic obstructive pulmonary disease and diabetes.

### 3.5. Distribution of Clinical and Molecular Features by Diagnostic Group

Table 5 shows the distribution of clinico-demographic, molecular, and occupational exposure status variables according to tumor histopathological subtype.

The distribution of patients by diagnostic group according to sex is shown in Table 5. Among females, 18 (13.7%) were included in G1a, 10 (7.6%) in G1b, 9 (6.9%) in G2, and 16 (12.2%) in G3. In male patients, the distribution was 27 (20.6%) in G1a, 19 (14.5%) in G1b, 21 (16.0%) in G2, and 11 (8.4%) in G3.

The chi-square test did not reveal a statistically significant association between patient sex and histopathological subtype (*p* = 0.124).

Although no significant sex differences in histological group distribution were observed, the data suggest a slight trend of predominance of G1a in males and G3 in females, with no clinical predictive value in this group.

The distribution of comorbidities showed significant differences between tumor groups (χ^2^ = 31.56, *p* = 0.016, Cramer’s V = 0.346, 95% CI [0.210–0.450]). Asthma was reported exclusively in the G1a group (6.1%), whereas COPD was found in all subtypes, but more frequently in G1b and G2 (9.2% and 9.9%, respectively). Heart disease and diabetes had a relatively balanced distribution between groups, but with slight variations (e.g., diabetes was more common in G1b and G2—3.8% and 4.6%, compared to 3.1% in G3).

The analysis of the distribution of TNM stages according to diagnostic group showed a relatively balanced distribution between the analyzed categories, with no statistically significant differences. The highest proportion of cases was concentrated in stage IV (overall 68.0%, 95% CI: 59.7–75.5%), with 31 patients in G1a (23.7%), 18 in G1b (13.7%), 22 in G2 (16.8%), and 18 in G3 (13.7%). In stage III, the distribution was more dispersed: 10 patients in G1a (7.6%), 9 in G1b (6.9%), 5 in G2 (3.8%), and 7 in G3 (5.3%). Stage II was predominantly represented in G1a and G2, with lower absolute frequencies (less than 5% of the total).

The chi-square test initially applied showed a non-significant value (χ^2^ = 1.886, *p* = 0.930), but given the partially violated validity conditions (33.3% of the cells had expected frequencies below 5), the variant with correction by Monte Carlo simulation was also applied. This confirmed the lack of statistically significant association between the two variables (*p* = 0.935, 99% confidence interval: 0.928–0.941).

The expression of the p63 marker was significantly different between the groups (χ^2^ = 33.92, *p* < 0.0001, Cramer’s V = 0.359, 95% CI [0.230–0.470]). While 29.8% of patients in G1a had positive expression, only 6.9% of G1b had this marker, confirming its predominant association with the G1a group. In the G2 group, p63 was positive in 15.3% of patients, and in G3 in 12.2%.

The PD-L1 status was also significantly associated with the diagnostic group (χ^2^ = 11.98, *p* = 0.007, Cramer’s V = 0.214, 95% CI [0.080–0.320]). In the G1a group 22.9% had positive expression, compared with only 5.3% in G3, suggesting a possible activation of the immune pathway in the G1a and G2 forms. Negative PD-L1 predominated in the G3 (15.3%) and G1b (9.2%) groups.

EGFR status did not differ significantly between groups (*p* = 0.365). The highest proportion of patients had negative status (no mutations)—32.1% in G1a, 20.6% in G2, 17.6% in G1b, and 15.3% in G3. Mutations detected on exons were rare, with no distinct pattern: exon 19 was present in six patients, exon 21 in five patients, and exons 18 and 20 in two–three cases each.

Regarding occupational exposure, no significant association was found between this variable and the diagnostic group (*p* = 0.853). The distributions were relatively homogeneous: in G1a, 18.3% of the patients were exposed, and in G1b and G2—12.2% and 13.7%, respectively. Unexposed cases had close weights in all groups.

This analysis confirms the significant association of p63 and PD-L1 expression with histopathological subtype, while other factors, such as TNM stage, EGFR, or occupational exposure, did not show relevant differences according to tumor group.

### 3.6. Analysis by Age Groups

For the analysis of clinical and molecular variables according to age, patients were divided into three conceptual classes based on biological assumptions related to occupational exposure and tumor latency. Thus, Class 1 included patients under 50 years of age, considered to be in the short or recent exposure stage, with a potentially insufficient latency interval for the development of complex tumors. Class 2 (50–64 years of age) was defined as the group of occupationally active adults with an increased likelihood of direct and sustained exposure to occupational risk factors. Finally, Class 3 (≥65 years) reflected the advanced post-exposure stage, in which the tumor process could manifest clinically as a result of the accumulation of effects over time.

This categorization was deliberately chosen to reflect the study’s main hypothesis that age could function as a biological proxy for the level and duration of occupational exposure and latency in the development of disease. Alternative classifications (based on statistical tertiles or quartiles) were tested exploratorily and did not reveal additional significant associations, which is why the biologically justified division was retained.

Table 6 shows the distribution of demographic, clinical, molecular, and occupational exposure variables according to the three age classes: Class 1 (<50 years), Class 2 (50–64 years), and Class 3 (≥65 years). The objective was to identify specific age-related patterns in the study population.

Smoking status showed no significant differences between the three groups (*p* = 0.678), although there was an increase in the proportion of smokers with age: from 12.2% in Class 1 to 25.2% in Class 2 and 31.3% in Class 3. In terms of background, the distribution of rural and urban patients was relatively balanced between classes, with no significant differences (*p* = 0.322).

Comorbidities were more frequently reported in the upper age classes, with 38.2% in Class 3, compared to 27.5% in Class 2 and 16.8% in Class 1. However, the chi-square test did not identify a statistically significant association between comorbidities and age class (*p* = 0.414).

The distribution of the histopathological groups (G1a–G3) was not significantly influenced by age (χ^2^ = 13.27, *p* = 0.149, Cramer’s V = 0.142, 95% CI [0.000–0.240]), although there was a slight predominance of G1a in Class 3 (18.3%) and G1b in Class 2 (9.2%). G3 was more frequent in Class 3 (10.7%) compared to the other categories.

The distribution of disease stage according to age group is also shown in Table 6. Among patients in Class 1 (<50 years), there were 3 cases in stage II (2.3%), 8 in stage III (6.1%), and 15 in stage IV (11.5%). For patients in Class 2 (50–64 years), 4 cases were reported in stage II (3.1%), 12 in stage III (9.2%), and 31 in stage IV (23.7%). In Class 3 (≥65 years), 4 cases were reported in stage II (3.1%), 11 in stage III (8.4%), and 43 in stage IV (32.8%).

The chi-square test with Monte Carlo correction did not reveal a statistically significant association between TNM stage and age class (χ^2^ = 2.384, df = 4, *p* = 0.681, 99% CI: 0.669–0.693).

Although a statistical difference between age classes was not demonstrated, a numerical trend was observed: the proportion of stage IV cases increased with age (from 11.5% in Class 1 to 32.8% in Class 3). However, this trend did not reach the threshold of statistical significance, including in the linear analysis (*p* = 0.156). This suggests that, in this group, age does not directly influence the severity of clinical stage at diagnosis.

The expression of the molecular markers p63 and PD-L1 did not vary significantly with age (*p* = 0.463 and *p* = 0.828, respectively). For p63, 26.0% of patients in Class 3 had positive expression, compared with 25.2% in Class 2 and 13.0% in Class 1. For PD-L1, the distribution of positive expression was similar: 24.4% in Class 3, 20.6% in Class 2, and 9.9% in Class 1.

EGFR status (mutant vs. negative) was not associated with age (*p* = 0.914). The highest proportion of EGFR-negative patients was in Class 3 (37.4%), followed by Class 2 (31.3%) and Class 1 (16.8%). EGFR-mutated cases were few, with no age distribution pattern.

The Ki-67 proliferation index was analyzed using the Kruskal–Wallis test and showed no statistically significant differences between age classes (*p* = 0.270), although the mean ranks were slightly higher in Class 3 (71.74) compared to Class 2 (59.83) and Class 1 (64.35).

No statistically significant association was observed between age group and TNM stage (chi-square test, *p* = 0.681), indicating that the differences in stage distribution across age categories are likely due to random variation rather than a true effect.

Finally, occupational exposure was not significantly associated with age (*p* = 0.919), although it was reported more frequently in Class 3 (26.0%) than in Class 2 (20.6%) and Class 1 (10.7%).

The only significant association observed in further analysis was between EGFR status and PD-L1 expression (*p* = 0.007). EGFR-positive patients had significantly more frequently negative PD-L1 expression (73.7% vs. 40.2% in EGFR-negative), suggesting a possible inverse relationship between the two molecular mechanisms.

### 3.7. Clinical–Molecular Composite Score: Definition, Rationale, and Stratification

To integrate relevant clinical and biological variables into a unified assessment framework, we developed an original composite score—EXPoSURE score (EXPosure-linked Severity and Underlying Risk Evaluation). It was designed to reflect disease severity and tumor aggressiveness in lung cancer patients by combining molecular markers with clinico-demographic indicators.

The score was initially calculated on a binary scale of 0–5 points, by allocating one point for each of the following characteristics present:

- Positive expression of PD-L1;
- Positive expression of p63;
- Confirmed mutation in the EGFR gene;
- Advanced clinical stage (TNM III or IV);
- Presence of comorbidities;
- Histological subtype with increased aggressiveness (microcellular carcinoma or neuroendocrine tumor).

In order to optimize the statistical robustness of the analysis in the context of a moderate sample (*n* = 131), the score was further ordinal recoded into three classes:

- Low score: 0–1 points;
- Medium score: 2–3 points;
- High score: 4–5 points.

This reclassification was motivated by the preliminary analysis, which indicated a limited discriminative ability of the continuous form of the score between different histopathological and molecular subgroups. The ordinal variable transformation resulted in a more balanced distribution and increased sensitivity in detecting statistically significant associations.

The EXPoSURE score was further utilized as an ordinal variable for testing associations with occupational exposure, biomarkers, and clinico-demographic characteristics, proposing itself as a potential multidimensional stratification tool in the clinical assessment of lung cancer patients.

The analysis of the distribution of the composite EXPoSURE score (EXPosure-linked Severity and Underlying Risk Evaluation), constructed to integrate relevant clinico-molecular characteristics, revealed a predominance of intermediate values in the study population. Of the 131 patients included, 78 (59.5%) fell into the medium score category, suggesting a mixed biological profile with elements of partial severity. Also, 39 patients (29.8%) presented a high score, indicating an association with clinical and molecular parameters of increased severity. Only 14 patients (10.7%) had a low score, reflecting a lower biological and tumor risk profile.

**Figure 3:**
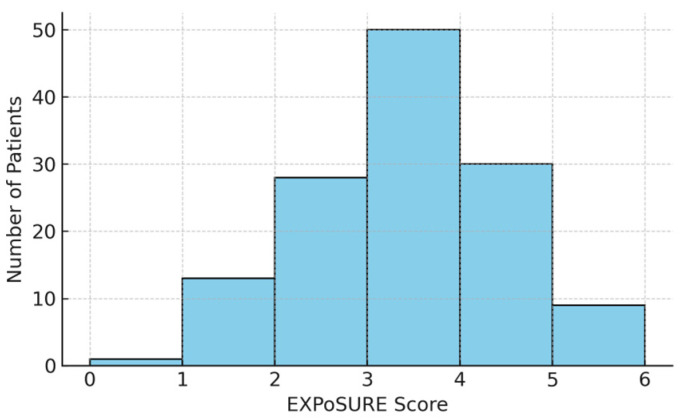
Distribution of EXPoSURE scores in the study cohort.

To further illustrate the distribution of EXPoSURE scores within the study cohort, we generated a histogram representing the frequency of each score value (Figure 3). The distribution demonstrates a predominance of intermediate values, with fewer patients at the extremes, reflecting the balanced contribution of the clinical and molecular parameters included in the score. This visual representation complements the statistical analyses and facilitates an intuitive understanding of the score’s discriminatory potential.

This distribution supports the applicability of the score as a stratification tool in analysing the relationship between occupational exposure and the patients’ oncological profile. At the same time, the increased proportion of medium and high scores confirms the heterogeneity of the group and justifies the exploration of associations with other clinico-pathological factors.

The EXPoSURE score distribution was analyzed in relation to the four diagnostic groups defined on the basis of histopathological and clinical criteria. The results show statistically significant differences between groups (χ^2^ = 14.352, df = 6, *p* = 0.026; *p* Monte Carlo = 0.024, 99% CI: 0.020–0.028), confirming a relevant association between score severity and diagnostic group.

In detail, patients in the G1a group frequently presented high (21 cases—16.0%) and medium (20 cases—15.3%) scores, indicating a predominantly severe distribution. The G1b group was dominated by medium scores (22 cases—16.8%) and had a low proportion of high scores (4 cases—3.1%). The G2 group maintained a balanced proportion between medium (18 cases—13.7%) and high scores (10 cases—7.6%), while the G3 group had the highest proportion of low scores (5 cases—3.8%) compared to the other groups.

These results suggest that the EXPoSURE score succeeds in capturing biological and severity differences between subgroups of patients, reinforcing its value in clinical stratification.

Analysis of the distribution of the EXPoSURE score according to histopathological tumor subtype revealed a statistically significant association (χ^2^ = 14.352, df = 6, *p* = 0.026, Cramer’s V = 0.264, 95% CI [0.073–0.343]), suggesting relevant differences between subtypes in the severity of the clinico-molecular features integrated in the score.

Patients with adenocarcinoma were predominantly categorized as medium (20 cases—15.3%) and high (21 cases—16.0%), suggesting a broad spectrum of biological aggressiveness. In contrast, in squamous carcinoma, the majority of patients had a medium score (22 cases—16.8%), and only 4 patients (3.1%) had a high score, suggesting a less severe profile.

As for microcellular carcinoma, the proportions were distributed between a medium score (18 cases—13.7%) and a high score (10 cases—7.6%), indicating a trend towards severity. Neuroendocrine tumors had a relatively balanced distribution between low, medium, and high scores, highlighting the diversity of biological behavior in this subgroup.

These results support the use of the EXPoSURE score as a tool to differentiate between histological types of lung cancer, suggesting that the score may reflect not only overall severity but also specific features of each tumor subtype.

Analyses of the association between EXPoSURE score level and PD-L1 expression status revealed a statistically significant difference (χ^2^ = 19.706, df = 2, *p* < 0.001, Cramer’s V = 0.376, 95% CI [0.267–0.476]). The results suggest a clear relationship between PD-L1 expression level and composite score severity.

Specifically, in the group of PD-L1-positive patients, almost half had a high score (32 cases—24.4% of the total), compared with only 7 cases (5.3%) with a high score among PD-L1-negative patients. In contrast, the majority of PD-L1-negative patients had low or medium scores, indicating a less aggressive biological profile.

This correlation reinforces the validity of the EXPoSURE score as an indicator of tumor activity and supports the integration of the PD-L1 marker into the score structure. Increased expression of PD-L1 appears to be associated with a more aggressive biological profile, directly reflected by higher scores.

The results revealed a statistically significant association between EXPoSURE score level and tumor marker p63 expression (χ^2^ = 24.353, df = 2, *p* < 0.001, Cramer’s V = 0.406, 95% CI [0.296–0.505]), suggesting an inverse relationship between the presence of p63 and the severity of the oncological profile assessed by this score.

More than 70% of the high-scoring patients were p63-positive (34 out of 39 cases), while only 5 patients (3.8%) with a high score had a negative expression. In the low-scoring group, the majority of patients were p63-negative (12 of 14 cases), emphasizing the tendency for high scores to be associated with the presence of the p63 marker.

This inversely proportional relationship between score level and absence of p63 expression provides further support for the biological validity of the EXPoSURE score. The presence of p63 appears to characterize subgroups with increased tumor activity, reflected by higher scores.

The analysis of the relationship between EXPoSURE score and EGFR mutational status (binary variance: negative vs. positive) revealed a statistically insignificant difference (χ^2^ = 4.171, df = 2, *p* = 0.124), although some relevant trends were observed at the descriptive level.

The majority of the high-scoring patients were EGFR-negative (37 out of 39 cases—28.2% of the overall total), and only 2 high-scoring patients (1.5%) had EGFR mutations. In the medium-scoring group, the proportion of mutations was slightly higher (15 positive cases—11.5%), but still in the minority.

Although the differences between the groups do not reach statistical significance, these observations suggest that the presence of EGFR mutations is more frequently associated with medium scores and rarely with high scores, indicating a possible distinct biological behavior. However, the findings should be interpreted with caution and further validation using larger samples is recommended.

There was a statistically significant association between EXPoSURE score level and TNM clinical TNM stage (χ^2^ = 22.605, df = 4, *p* < 0.001, Cramer’s V = 0.520, 95% CI [0.413–0.607]), indicating that the score faithfully reflects clinical tumor progression.

Specifically, of the high-scoring patients, 94.9% (37 out of 39 cases) were stage IV, suggesting a direct association between high scores and disease progression. In contrast, low-scoring patients were predominantly found in stages II and III (8 out of 14 cases), and only 5 (3.8%) low-scoring cases were in stage IV.

The gradual distribution of the score according to TNM stage reinforces its value as an integrative marker of severity, able to distinguish between early and advanced forms of the disease. These data support the use of the EXPoSURE score in comprehensive clinical risk assessment.

**Figure 4:**
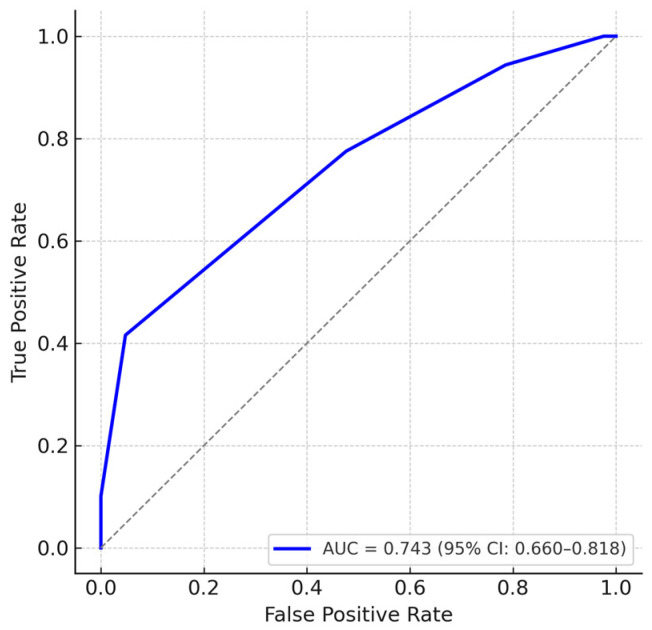
ROC curve for the EXPoSURE score in predicting stage IV disease (AUC = 0.743, 95% CI: 0.660–0.818).

To further assess the discriminatory ability of the EXPoSURE score, we performed a receiver operating characteristic (ROC) analysis using stage IV disease as the reference outcome. The score achieved an area under the curve (AUC) of 0.743 (95% CI: 0.660–0.818), indicating good ability to differentiate advanced-stage from non-advanced-stage patients. Internal validation using bootstrap resampling with 1000 iterations confirmed the stability of the AUC estimate, with minimal optimism (corrected AUC = 0.740). An additional ROC analysis was conducted considering advanced disease as TNM stages III–IV versus non-advanced stages I–II. This yielded an AUC of 0.657 (95% CI: 0.569–0.737), suggesting moderate discriminatory performance and supporting the applicability of the score across different definitions of advanced disease (Figure 4).

The results showed a strong statistically significant association between the EXPoSURE score level and occupational exposure history (χ^2^ = 29.772, df = 2, *p* < 0.001, Cramer’s V = 0.430, 95% CI [0.322–0.529]), confirming the central hypothesis of the study on the influence of occupational exposure on tumor biology profile.

Of the high-scoring patients, the overwhelming majority (34 of 39 cases—87.2%) had a history of occupational exposure. In contrast, patients with no exposure had mainly low (13 cases—9.9%) or medium (38 cases—29.0%) scores, and only 5 patients (3.8%) in this group had high scores.

These data reinforce the relevance of the EXPoSURE score in assessing the cumulative effects of occupational environmental factors on the severity and aggressiveness of oncological disease. Thus, the score can be considered not only as a marker of tumor severity, but also as an indirect indicator of the impact of chronic exposure to occupational hazards on the etiology and progression of the disease (Table 7).

## 4. Discussion

The originality of this study stems from its exclusive reliance on bronchoscopy and EBUS-TBNA for both the diagnosis and, in part, the staging of lung cancer, thereby avoiding the need for surgical thoracic procedures. In Romania, particularly in the Oltenia region, the systematic implementation of EBUS-TBNA had not been previously documented, which underscores the novelty and potential relevance of this research for regional clinical practice.

Our findings confirmed that lung cancer remained more prevalent among male patients, although recent studies indicate that this gender gap is narrowing, likely due to similar smoking exposure across sexes [22]. Smoking was strongly associated with most histological types of lung cancer, supporting evidence that it accounts for approximately 85–90% of cases worldwide [23]. Notably, adenocarcinoma emerged as the most common histological type among non-smoking women in our sample, aligning with global reports that this subtype has become a leading cause of cancer-related deaths in never-smoking females [24]. Occupational exposure also played a role across all lung cancer subtypes, echoing findings from studies linking workplace pollutants and lung cancer risk [25].

In this cohort, 68% of patients were diagnosed in stage IV according to the current TNM classification (eighth edition), reflecting silent clinical progression and a lack of systematic screening programs. These findings mirror data from Canada, where about 50% of lung cancer cases are detected at stage IV [26], and from China, where a similar predominance of advanced-stage diagnoses has been reported, especially among male patients [27].

Regarding diagnostic procedures, EBUS-TBNA demonstrated an impressive diagnostic yield with a low rebiopsy rate of 8.4% and no requirement for mediastinoscopy. Recent literature confirms its high diagnostic accuracy and its ability to reduce the need for surgical confirmation [12,13]. Our study also validated EBUS-TBNA’s capacity to provide adequate samples for both histopathology and molecular testing, consistent with previous systematic reviews [28,29].

Although molecular markers such as PD-L1, p63, and EGFR are valuable for therapeutic decision-making, they did not show statistically significant correlations with TNM stage in our cohort. Similar observations have been reported by Miyamoto et al. [30] and Ilie et al. [31]. This could reflect both the heterogeneity of our sample and the limited role of these biomarkers in anatomical lymph node staging. Nonetheless, their inclusion remains critical for guiding targeted therapies.

In light of these findings, we developed a clinical–molecular composite score (EXPoSURE) that integrates PD-L1 expression, p63, EGFR status, comorbidities, histological subtype, and TNM stage. This score was designed as a synthetic instrument to reflect oncological severity, particularly in relation to the influence of occupational exposure. Its reclassification into a three-tier ordinal system allowed clearer subgroup differentiation, suggesting its value for clinical risk stratification.

Conceptually, the EXPoSURE score resembles other multidimensional indices, such as the lung immune prognostic index (LIPI) [32] or the SCOUT score [33], which combine clinical and molecular parameters to refine risk assessment in lung cancer. Such integrative models have been shown to outperform single-parameter tools [29], further supporting the rationale for a composite scoring approach in this study. The lung immune prognostic index (LIPI) has been validated across multiple NSCLC cohorts and in meta-analyses as a robust prognostic marker, while other composite scores such as SCOUT integrate diverse clinical and biomarker data to improve patient stratification. Including such references reinforces the rationale for developing multidimensional tools like the EXPoSURE score.

In addition to the correlation analyses, the EXPoSURE score was evaluated using ROC curve analysis, which demonstrated a good discriminative performance for identifying stage IV disease (AUC = 0.743, 95% CI: 0.660–0.818). While these results support the score’s potential clinical utility, they also highlight the need for prospective validation in larger and more diverse cohorts.

Although the EXPoSURE score was not initially designed as a prognostic or therapeutic tool, it may offer a more nuanced perspective on the biological complexity of lung cancer.

The secondary objective of this study focused on exploring the potential influence of occupational exposure on patients’ clinical and molecular profiles. Although no statistically significant differences emerged when analyzing individual variables, the EXPoSURE score showed suggestive trends associated with occupational history. This suggests that chronic occupational exposure may contribute to a more severe clinical profile and complex systemic alterations, which are difficult to capture through single biomarkers alone.

Although the current data do not permit definitive conclusions, this line of investigation remains highly relevant for future research and could serve as a basis for dedicated prospective studies.

Several limitations of this study should be acknowledged. First, its retrospective, single-center design may compromise both internal and external validity, introducing potential selection bias and recording errors, as highlighted by Kundu et al. [34]. Second, the relatively small sample size (*n* = 131) and the uneven subgroup distribution could affect statistical power, a known challenge in clinical research that requires robust a priori sample size estimations [35]. Moreover, longitudinal follow-up data on survival and treatment response were not available for the present retrospective cohort, which precluded correlation analyses between these endpoints and the EXPoSURE score. Inclusion of these clinically relevant outcomes is planned in a prospective validation study to more comprehensively assess the prognostic value of the score. Additionally, the lack of long-term survival data prevents the evaluation of the prognostic capacity of the EXPoSURE score. Finally, relying on self-reported occupational histories without quantitative exposure measurements introduces a risk of misclassification and confounding, which is common in retrospective studies. In addition, occupational exposure was classified only as present or absent, without specifying the type of occupational agents, which may further limit the precision of the exposure assessment.

Another limitation relates to the statistical analysis. No formal adjustments for multiple testing were performed, given the exploratory nature of the study and its objective to generate hypotheses for further research. Consequently, statistically significant associations should be interpreted with caution, and confirmation in larger, independent cohorts is warranted.

Future studies will aim to address the current limitations by collecting detailed data on various types of occupational exposure, including the duration and intensity levels. This approach will enable a more precise correlation between these exposure characteristics and the severity of diseases. Additionally, a prospective multicenter validation of the EXPoSURE score in larger cohorts is planned to confirm its applicability and enhance subgroup analyses, particularly concerning the effects of occupational exposure.

Furthermore, upcoming research will focus on integrating additional molecular markers and refining the score’s ability to distinguish between different outcomes, as indicated by the ROC analysis in this study. Prospective designs that assess the score’s predictive value regarding treatment response and survival could help establish the EXPoSURE score as a clinically relevant tool for personalized management and risk stratification in lung cancer. Taken together, these findings emphasize the clinical value of EBUS-TBNA in lung cancer staging, offering an effective and safe alternative to more invasive surgical methods. While individual molecular markers did not significantly differentiate TNM stages, their integration into the EXPoSURE composite score enabled a multidimensional stratification tool with potential clinical utility. Considering occupational exposure as part of this integrative approach may add relevant insight into the oncological profile of patients and justify further investigation.

Validation of the EXPoSURE score in larger, multicenter prospective cohorts, along with survival and treatment–response correlations, is needed. Prospective studies may also help clarify the real impact of occupational exposure on clinico-molecular severity. Beyond the inherent limitations of a retrospective design, these results support the rationale for an integrated approach to lung cancer patient staging and characterization with practical applicability in contemporary oncological practice.

## 5. Conclusions

This study confirms the diagnostic and staging value of EBUS-TBNA in patients with lung cancer, allowing minimally invasive assessment and reducing the need for surgical procedures. While individual molecular markers did not show significant associations with TNM stages, the integration of clinical and molecular parameters into the EXPoSURE composite score offered a promising approach to multidimensional patient evaluation. Although exploratory, the observed trends related to occupational exposure highlight the potential role of environmental factors in shaping disease severity. Further multicenter prospective studies are warranted to validate the EXPoSURE score and to better understand its prognostic implications.

## Figures and Tables

**Table 1:** Distribution of patients’ demographic and clinical characteristics by gender.

Parameter	Value	Gender		*p*
		Female	Male	
Residential environment	rural	19 (14.5%)	35 (26.7%)	0.303 *
	urban	34 (26.0%)	43 (32.8%)	
Smoker status	NO	21 (16.0%)	20 (15.3%)	0.090 *
	YES	32 (24.4%)	58 (44.3%)	
Comorbidities	None	9 (6.9%)	14 (10.7%)	0.002 *^#^
	Asthma	8 (6.1%)	0 (0.0%)	
	COPD	20 (15.3%)	28 (21.4%)	
	Heart disease	9 (6.9%)	14 (10.7%)	
Occupational exposure	NO	23 (17.6%)	33 (25.2%)	0.902 *
	YES	30 (22.90%)	45 (34.4%)	

* Chi-square test. # Statistically significant.

**Table 2:** Patient characteristics by background: smoking, comorbidities, and occupational exposure.

Parameter	Value	Residential Environment		*p*
		Rural	Urban	
Smoker status	NO	16 (12.20%)	25 (19.10%)	0.730 *
	YES	38 (29.0%)	52 (39.70%)	
Comorbidities	None	12 (9.2%)	11 (8.4%)	0.391 *
	Asthma	1 (0.8%)	7 (5.3%)	
	COPD	19 (14.5%)	29 (22.1%)	
	Heart disease	13 (9.9%)	16 (12.2%)	
Occupational exposure	NO	9 (6.9%)	14 (10.7%)	0.976 *
	YES	23 (17.6%)	33 (25.2%)	

* Chi-square test.

**Table 3:** Smoking status and comorbidities in relation to occupational exposure.

Parameter	Value	Occupational Exposure		*p*
		NO	YES	
Smoker status	NO	20 (15.3%)	21 (16.0%)	0.346 *
	YES	36 (27.5%)	54 (41.2%)	
Comorbidities	None	9 (6.9%)	14 (10.7%)	0.491 *
	Asthma	5 (3.8%)	3 (2.3%)	
	COPD	17 (13.0%)	31 (23.7%)	
	Heart disease	15 (11.5%)	14 (10.7%)	
	Diabetes	10 (7.6%)	13 (9.9%)	

* Chi-square test.

**Table 4:** Distribution of comorbidities by smoking status in the study cohort.

Parameter	Value	Smoker Status		*p*
		NO	YES	
Comorbidities	None	5 (3.8%)	18 (13.7%)	<0.001 *^#^
	Asthma	8 (6.1%)	0 (0.0%)	
	COPD	14 (10.7%)	34 (26.0%)	
	Heart disease	11 (8.4%)	18 (13.7%)	
	Diabetes	3 (2.3%)	20 (15.3%)	

* Chi-square test (Monte Carlo). # Statistically significant.

**Table 5:** Distribution of clinical and molecular parameters according to diagnostic group.

Parameter	Value	Diagnostic Group				*p*
		G1a	G1b	G2	G3	
Gender	Female	18 (13.7%)	10 (7.6%)	9 (6.9%)	16 (12.2%)	0.124 *
	Male	27 (20.6%)	19 (14.5%)	21 (16.0%)	11 (8.4%)	
Comorbidities	None	5 (3.8%)	3 (2.3%)	6 (4.6%)	9 (6.9%)	0.016 *^#^
	Asthma	8 (6.1%)	0 (0.0%)	0 (0.0%)	0 (0.0%)	
	COPD	16 (12.2%)	12 (9.2%)	13 (9.9%)	7 (5.3%)	
	Heart disease	8 (6.1%)	9 (6.9%)	5 (3.8%)	7 (5.3%)	
	Diabetes	8 (6.1%)	5 (3.8%)	6 (4.6%)	4 (3.1%)	
TNM stage	II	4 (3.1%)	2 (1.5%)	3 (2.3%)	2 (1.5%)	0.935 **
	III	10 (7.6%)	9 (6.9%)	5 (3.8%)	7 (5.3%)	
	IV	31 (23.7%)	18 (13.7%)	22 (16.8%)	18 (13.7%)	
P63	Negative	6 (4.6%)	20 (15.3%)	10 (7.6%)	11 (8.4%)	<0.0001 *^#^
	Positive	39 (29.8%)	9 (6.9%)	20 (15.3%)	16 (12.2%)	
PD-L1	Negative	15 (11.5%)	12 (9.2%)	12 (9.2%)	20 (15.3%)	0.007 *^#^
	Positive	30 (22.9%)	17 (13.0%)	18 (13.7%)	7 (5.3%)	
EGFR	None	42 (32.1%)	23 (17.6%)	27 (20.6%)	20 (15.3%)	0.365 *
	exon18	0 (0.0%)	1 (0.8%)	0 (0.0%)	1 (0.8%)	
	exon19	1 (0.8%)	2 (1.5%)	3 (2.3%)	3 (2.3%)	
	exon20	0 (0.0%)	1 (0.8%)	0 (0.0%)	2 (1.5%)	
	exon21	2 (1.5%)	2 (1.5%)	0 (0.0%)	1 (0.8%)	
Occupational exposure	NO	21 (16.0%)	13 (9.9%)	12 (9.2%)	10 (7.6%)	0.853 *
	YES	24 (18.3%)	16 (12.2%)	18 (13.7%)	17 (13.0%)	

* Chi-Square Tests, ** Chi-square test (Monte Carlo). # Statistically significant.

**Table 6:** Distribution of demographic, clinical, and molecular characteristics by age group.

Parameter	Value	Age Group			*p*
		<50 Years	50–64 Years	64< Years	
Gender	Female	13 (9.9%)	18 (13.7%)	22 (16.8%)	0.5416 *
	Male	13 (9.9%)	29 (22.1%)	36 (27.5%)	
Smoking status	NO	10 (7.6%)	14 (10.7%)	17 (13.0%)	0.678 *
	YES	16 (12.2%)	33 (25.2%)	41 (31.3%)	
Residential environment	Rural	14 (10.7%)	17 (13.0%)	23 (17.6%)	0.322 *
	Urban	12 (9.2%)	30 (22.9%)	35 (26.7%)	
Comorbidities	NO	4 (3.1%)	11 (8.4%)	8 (6.1%)	0.414 *
	YES	22 (16.8%)	36 (27.5%)	50 (38.2%)	
Diagnostic group	G1a	5 (3.8%)	16 (12.2%)	24 (18.3%)	0.149 *
	G1b	9 (6.9%)	12 (9.2%)	8 (6.1%)	
	G2	5 (3.8%)	13 (9.9%)	12 (9.2%)	
	G3	7 (5.3%)	6 (4.6%)	14 (10.7%)	
TNM stage	II	3 (2.3%)	4 (3.1%)	4 (3.1%)	0.681 **
	III	8 (6.1%)	12 (9.2%)	11 (8.4%)	
	IV	15 (11.5%)	31 (23.7%)	43 (32.8%)	
p63	Negative	9 (6.9%)	14 (10.7%)	24 (18.3%)	0.463 *
	Positive	17 (13.0%)	33 (25.2%)	34 (26.0%)	
PD-L1	Negative	13 (9.9%)	20 (15.3%)	26 (19.8%)	0.828 *
	Positive	13 (9.9%)	27 (20.6%)	32 (24.4%)	
EGFR	Negative	22 (16.8%)	41 (31.3%)	49 (37.4%)	0.914 *
	Positive	4 (3.1%)	6 (4.6%)	9 (6.9%)	
Occupational exposure	NO	12 (9.2%)	20 (15.3%)	24 (18.3%)	0.919 *
	YES	14 (10.7%)	27 (20.6%)	34 (26.0%)	

* Chi-Square Tests, ** Chi-square test (Monte Carlo).

**Table 7:** Distribution of the EXPoSURE composite score in relation to clinical and molecular parameters.

Parameter	Value	EXPoSURE Score			*p*
		Low Score	Medium Score	High Score	
Diagnostic group	G1a	4 (3.1%)	20 (15.3%)	21 (16.0%)	0.026 **^#^
	G1b	3 (2.3%)	22 (16.8%)	4 (3.1%)	
	G2	2 (1.5%)	18 (13.7%)	10 (7.6%)	
	G3	5 (3.8%)	18 (13.7%)	4 (3.1%)	
Comorbidities	NO	2 (1.5%)	13 (9.9%)	8 (6.1%)	0.826 *
	YES	12 (9.2%)	65 (49.6%)	31 (23.7%)	
Histopathological subtype	Adenocarcinoma	4 (3.1%)	20 (15.3%)	21 (16.0%)	0.026 **^#^
	Squamous cell carcinoma	3 (2.3%)	22 (16.8%)	4 (3.1%)	
	Small cell carcinoma	2 (1.5%)	18 (13.7%)	10 (7.6%)	
	Pulmonary neuroendocrine tumor	5 (3.8%)	18 (13.7%)	4 (3.1%)	
TNM stage	II	3 (2.3%)	7 (5.3%)	1 (0.8%)	<0.001 *^#^
	III	6 (4.6%)	24 (18.3%)	1 (0.8%)	
	IV	5 (3.8%)	47 (35.9%)	37 (28.2%)	
p63	Negative	12 (9.2%)	30 (22.9%)	5 (3.8%)	<0.001 *^#^
	Positive	2 (1.5%)	48 (36.6%)	34 (26.0%)	
PD-L1	Negative	11 (8.4%)	41 (31.3%)	7 (5.3%)	<0.001 *^#^
	Positive	3 (2.3%)	37 (28.2%)	32 (24.4%)	
EGFR	Negative	12 (9.2%)	63 (48.1%)	37 (28.2%)	0.124 *
	Positive	2 (1.5%)	15 (11.5%)	2 (1.5%)	
Occupational exposure	NO	13 (9.9%)	38 (29.0%)	5 (3.8%)	<0.001 *^#^
	YES	1 (0.8%)	40 (30.5%)	34 (26.0%)	

* Chi-Square Tests, ** Chi-square test (Monte Carlo). # Statistically significant.

## Data Availability

The authors declare that the data of this research are available from the corresponding authors upon reasonable request.

## Abbreviations

The following abbreviations are used in this manuscript:

EBUS-TBNA	Endobronchial Ultrasound-Guided Transbronchial Needle Aspiration
NSCLC	Non-Small Cell Lung Cancer
SCLC	Small Cell Lung Cancer
PD-L1	Programmed Death Ligand 1
EGFR	Epidermal Growth Factor Receptor
ALK	Anaplastic Lymphoma Kinase
TNM	Tumor, Nodes, Metastases Staging System
CT	Computed Tomography
COPD	Chronic Obstructive Pulmonary Disease
